# Advancing Anemia Management in Chronic Kidney Disease: Assessing the Superiority of Darbepoetin Alfa Over Erythropoietin Alpha

**DOI:** 10.7759/cureus.51613

**Published:** 2024-01-03

**Authors:** Dinesh Khullar, Snehal S Muchhala, Abhishek T

**Affiliations:** 1 Nephrology and Renal Transplant Medicine, Max Super Specialty Hospital, New Delhi, IND; 2 Medical Affairs, Dr. Reddy’s Laboratories, Hyderabad, IND

**Keywords:** cost-effectiveness, anaemia management, chronic kidney disease (ckd), erythropoietin alfa, darbepoetin alfa

## Abstract

Anemia is a prevalent and debilitating complication in patients with chronic kidney disease (CKD). It presents multifaceted challenges that impact patients' quality of life and overall well-being. The advent of darbepoetin alfa (DPO) as a therapeutic alternative to recombinant human erythropoietin alpha (EPO) has revolutionized the management of CKD-associated anemia. This review article presents a comprehensive comparative analysis highlighting the advantages of DPO over EPO in the effective management of anemia, in both predialysis and dialysis-dependent (DD) CKD patients. DPO's distinct pharmacokinetic advantages play a pivotal role in its efficacy and safety. With a significantly longer half-life and several-fold increased biological activity compared to EPO, DPO enables extended dosing intervals. Through an in-depth examination of diverse clinical trials, it becomes evident that DPO consistently demonstrates remarkable efficacy and safety in improving and maintaining hemoglobin (Hb) levels. Furthermore, its simplified dosage regimen, coupled with the convenience of less frequent administration, not only improves patient adherence but also translates to reduced healthcare costs and resource utilization. In conclusion, this review provides compelling evidence of the advantages of DPO over conventional recombinant human EPO for managing anemia in CKD patients, both in the predialysis and dialysis-dependent CKD patients. DPO's pharmacokinetic advantages, patient-centered advantages, enhanced compliance, and cost-effectiveness converge to establish DPO as a transformative therapeutic option. In both predialysis and dialysis settings, DPO's superior efficacy and patient-centric attributes collectively redefine the landscape of anemia management in CKD.

## Introduction and background

Anemia is a very prevalent complication of chronic kidney disease (CKD), exerting a substantial impact on morbidity and mortality [1,2]. According to the Kidney Disease: Improving Global Outcomes (KDIGO) clinical practice guidelines, anemia of CKD is defined by hemoglobin (Hb) levels below 13.0 g/dL for men and below 12.0 g/dL for nonpregnant women [3]. The prevalence of anemia in CKD patients is markedly high and exhibits regional variation. Notably, studies have reported varying rates of anemia in CKD across different countries, such as 14% in the United States [4], 39.36% in India [5], and 51.5% in China [6]. A recent cross-sectional, prospective observational study conducted in India in 2021 reported an astounding prevalence of anemia among CKD patients, reaching 82.4% among the study participants [7]. Furthermore, the prevalence of anemia increases with the progression of CKD stages, with overall rates of 22.4%, 41.3%, and 53.9% in stages 3, 4, and 5 of CKD, respectively [8].

The etiology of anemia in CKD patients is intricate and multifactorial; however, inadequate production of erythropoietin alpha (EPO) by the failing kidneys stands as the predominant cause, resulting in disproportionately low levels of circulating EPO relative to the severity of anemia [9]. CKD is recognized as a potential contributor to anemia when the glomerular filtration rate falls below 60 mL/minute/1.73 m^2^ [3,10]. Extensive clinical evidence has consistently demonstrated a direct correlation between the severity of anemia and the decline in kidney function [11].

Anemia represents an independent risk factor for the development of left ventricular hypertrophy, heart failure, cardiovascular mortality, and an increased likelihood of progressing to end-stage renal disease (ESRD) [12-14]. Additionally, anemia of CKD contributes to fatigue, depression, and sleep disturbances [15,16]. Notably, anemia emerges as the complication with the most substantial impact on perceived quality of life, both in patients with CKD receiving hemodialysis (HD) and those with pre-dialysis CKD.

The correction of anemia is therefore imperative in CKD management. The KDIGO recommends iron supplementation as the first-line therapy in CKD patients with anemia and absolute or functional iron deficiency [3]. If anemia is not corrected after adequate iron supplementation, treatment with erythropoiesis-stimulating agents (ESAs) has demonstrated remarkable efficacy in correcting the Hb levels, reducing the requirement for blood transfusions, hospital admissions, and overall mortality [9,17-19]. Moreover, the beneficial effects of anemia correction extend to various aspects of patient well-being. These include improvements in quality of life (QOL), nutritional status, exercise tolerance, sexual function, glucose metabolism, and amelioration of clotting dysfunctions [20].

## Review

Exploring the potential of erythropoiesis-stimulating agents and the need for innovative molecules

In light of the adverse outcomes of anemia in CKD and the desire to circumvent the need for red blood cell transfusions, the standard approach involves correcting anemia through the administration of iron supplementation and ESAs [21]. Erythropoietin is a naturally occurring hormone that serves as the primary regulator of erythropoiesis. r-HuEPO is a glycoprotein that closely resembles endogenous human erythropoietin in terms of its biological activity and physical characteristics. Its introduction in the late 1980s revolutionized the management of anemia in CKD patients and has since been employed as the standard treatment for renal anemia for over four decades [22].

The use of ESAs, such as r-HuEPO, has been widespread in both dialysis-dependent and non-dialysis-dependent CKD patients to elevate Hb levels. Replacement therapy with r-HuEPO has proven to be an effective approach in the treatment of renal anemia. Apart from the well-established antiapoptotic effects, r-HuEPO has also shown the potential to stimulate regenerative cells, thereby contributing to tissue protection and regeneration in CKD patients [23].

However, a significant challenge associated with the use of r-HuEPO is the need for frequent administration, typically three times per week, due to its short half-life. While intravenous (IV) administration is feasible for patients undergoing HD, subcutaneous (SC) injections can be both frequent and painful, which can negatively impact treatment adherence in patients on peritoneal dialysis (PD) and pre-dialysis patients [24]. To maximize the therapeutic effectiveness of r-HuEPO, it is often necessary to administer the medication SC or IV two to three times per week. Consequently, researchers have explored the development of a new compound with a higher number of carbohydrate-containing side chains than r-HuEPO. The rationale behind this approach is that a molecule with an increased number of side chains would hypothetically exhibit a longer serum half-life, resulting in less frequent administration [24].

EPO and darbepoetin alfa (DPO) are two ESAs commonly utilized interchangeably for managing anemia in CKD patients, including those with ESRD necessitating dialysis. Numerous studies, including a considerable number of randomized controlled trials, have consistently demonstrated the effectiveness of both EPO and DPO in treating anemia associated with CKD.

Darbepoetin alfa versus erythropoietin alpha: unveiling the molecular secrets behind extended dosage intervals

Darbepoetin alfa (DPO), a second-generation long-acting ESA, is a newer alternative that elicits a physiological response similar to that of r-HuEPO [25]. DPO is an EPO analogue that undergoes hyperglycosylation to extend its survival in the bloodstream. In terms of the erythropoiesis mechanism, DPO shares similarities with endogenous EPO and EPO (rHuEPO). However, DPO distinguishes itself with its unique properties, including a prolonged half-life in the blood and increased erythropoietic activity compared to endogenous EPO and r-HuEPO [26,27].

DPO exhibits an approximately three-fold longer mean terminal half-life compared to r-HuEPO, which allows DPO to remain in circulation for a prolonged duration, enabling it to bind, activate, and interact with the erythropoietin receptor (EPO-R) for an extended period [28,29]. Hence, DPO can be administered at a reduced frequency compared to standard rHuEPO (EPO) while effectively sustaining sufficient serum concentrations to elicit erythropoiesis and achieve target Hb levels. These extended administration intervals represent an opportunity to simplify treatment and improve the efficiency of anemia management. The other advantages include two to three lower clearance of DPO than that of r-HuEPO, a 4.3-fold lower affinity for the EPO-R compared to recombinant human erythropoietin with DPO being 3.6-fold more potent than EPO [28,30]. Comparative features of various ESAs have been depicted in Table 1 [31-34].

**Table 1:** Comparative features of erythropoiesis-stimulating agents ^a^0.5×/week = once every two weeks. ^b^As assessed from animal studies. ^c^Depending on the dose of erythropoietin alpha (up to 33,999 U/week). h = hour; IV = intravenous; SC = subcutaneous; ND = no data; ×/week = times per week.

Parameters	Erythropoietin alpha	Erythropoietin beta	Darbepoetin alfa
Carbohydrate proportion (%)	40	40	52
Number of N-linked carbohydrates	3	3	5
Number of sialic acid residues per molecule	<14	<14	<22
Half-life (h): IV route	4–11	8.8–10.4	18–25.3
Half-life (h): SC route	19–25.3 (same in both predialysis and dialysis)	24 (same in both predialysis and dialysis)	48.8 in dialysis, 70 in predialysis
Clearance (IV route) (mL/h^–1^/kg^–1^)	8.1–8.6	7.9	2.0
Bioavailability (SC route) (%)	20–30	20	37
Frequency of administration (×/week)	1–3	0.5^a^–3	0.5^a^–1
Relative potency of thrice weekly dosing^b^	1	1–1.2	3.6
Relative potency of once weekly dosing^b^	1	ND	13–20
Conversion factor	1	1	200 IU : 1 μg (up to 433 : 1^c^)

The combination of DPO's prolonged half-life and high biological activity supports its utilization in less frequent dosing regimens, such as once weekly (QW) or extended intervals like once every other week (Q2W), to maintain Hb levels in patients with renal anemia. These findings highlight the potential advantages of utilizing DPO in optimized dosing schedules to effectively manage anemia in patients with CKD.

r-HuEPO is an acidic sialylglycoprotein hormone composed of a single chain comprising 165 amino acids. It possesses three N-linked and one O-linked oligosaccharide chains [35,36]. The sialic acid-containing carbohydrate content of ESAs has been shown to be associated with their receptor-binding affinities, circulating half-lives, and biological activities. EPO isoforms that possess a higher number of sialic acid residues exhibit reduced receptor-binding affinity but extended half-lives and increased biological activity [30,37]. In the case of DPO, it features a distinct structure compared to EPO, both in terms of its amino acid sequence and its carbohydrate content. These structural differences contribute to the unique pharmacokinetic and pharmacodynamic properties exhibited by DPO.

DPO exhibits five changes in the amino acid sequence compared to the native EPO molecule. Through site-directed mutagenesis of the HuEPO gene, modifications were made to create a new glycoprotein, i.e., DPO with two additional sialic acid-containing N-linked carbohydrate chains. In contrast, both the native EPO molecule and r-HuEPO have three such chains. This modification allows DPO to potentially have up to eight additional sialic acid residues [38]. The increased carbohydrate content of DPO compared to EPO leads to reduced binding affinity for the EPO-R. However, the longer half-lives of DPO result in enhanced biological activity [30,37,39].

Optimal dosing strategies while switching from EPO to DPO

The commonly used conversion ratio for switching patients from EPO to DPO is 200 international units (IU) of EPO to 1 μg of DPO. Several studies have found that when transitioning from EPO to DPO, a mean dose reduction ranging from 17% to 39% is necessary to maintain stable Hb levels even after converting from EPO to DPO using the traditional 200:1 ratio [9,40,41]. Table 2 shows the estimated starting dose of DPO in patients with CKD on dialysis while switching from EPO.

**Table 2:** Estimated darbepoetin alfa starting doses for patients with chronic kidney disease on dialysis based on previous erythropoietin alpha Adapted from the Aranesp package insert [42].

Previous weekly erythropoietin alpha dose (units/week)	Darbepoetin alfa dose (μg/week)
<2500	6.25
2500–4999	12.5
5000–10,999	25
11,000–17,999	40
18,000–33,999	60
34,000–89,999	100
>90,000	200

The advantage of DPO over EPO in terms of lower dosage requirements may be more pronounced in patients who require higher dosages of ESAs. This is supported by reports indicating higher conversion ratios for EPO compared to DPO in patients receiving higher ESA dosages [31].

Darbepoetin alfa versus erythropoietin alpha: a comparative analysis of clinical trials for effective anemia management in chronic kidney disease

In the realm of CKD management, the search for optimal anemia control has been an enduring challenge. EPO therapies have long been the cornerstone, but recent advances have ushered in a novel era marked by DPO as an intriguing alternative. A comprehensive scrutiny of clinical trials exploring DPO's efficacy vis-à-vis EPO in CKD predialysis and HD patients reveals an insightful narrative of therapeutic superiority (Table 3). These trials, characterized by their prospective nature and rigorous methodology, unveil compelling evidence of DPO's distinct advantages in anemia management, ranging from enhanced Hb elevation, reduced variability, and fewer dose adjustments to improved patient tolerability and simplified dosing regimens. By embarking on this journey through the clinical trial evidence, we gain invaluable insights into the transformative potential of DPO, offering a paradigm shift in the landscape of CKD anemia therapeutics.

**Table 3:** Comparative clinical trials data of darbepoetin alfa once a week versus erythropoietin alpha thrice weekly

Study	Patient	Key study findings
Lullo et al. [43]	Predialysis CKD patients	DPO exhibited significantly greater effectiveness in increasing Hb levels in comparison to EPO over the course of 3 (p < 0.0001), 6 (p < 0.0001), 9 (p < 0.01), and 12 months (p < 0.01); Hb target level of >11 g/dL was fully achieved for all patients treated with DPO, and only 70% of those treated with EPO (p < 0.01). Significant improvement in cardiovascular performance, specifically with regard to left ventricular end-diastolic volume, ejection fraction, and pro-BNP levels, was observed following the administration of DPO compared to EPO.
Mehta et al. [44]	Pre-dialysis CKD patients	Mean change in Hb from baseline to the end of the study was similar in both the DPO and EPO groups.
Bernieh et al. [45]	HD patients	Target Hb was successfully attained in 64.8% of the DPO-treated group compared to 59.7% in the EPO-treated group (p = 0.006). Hb variability was observed to be less frequent in the DPO group (p = 0.2), and incidents of vascular access thrombosis were significantly lower in the DPO group (1) compared to the EPO group (p < 0.001).
El-Ashmawy et al. [46]	HD patients	Noteworthy disparity in Hb levels between the two treatment groups, DPO and EPO, with a statistically significant mean difference of 0.77 g/dL (p < 0.0001); a significantly higher proportion of patients attaining the target Hb level of ≥ 11 g/dL within the DPO-treated group (84.6%) in comparison to the EPO group (51.9%) (p < 0.001).
Alkatheri et al. [47]	HD patients	Administration of various doses of DPO at 40, 60, 80, and 100 μg once a week elicited a substantial and consistently remarkable elevation in Hb levels, red blood cell (RBC) count, and hematocrit (Hct). Conversely, this favorable response was not mirrored in the group treated with EPO.
Kotb et al. [48]	HD patients	Mean Hb levels observed for the DPO and EPO groups were 11.75 g/dL and 10.98 g/dL, respectively. The calculated mean difference of 0.77 g/dL between these two treatment modalities proved to be statistically significant, (p <0.0001); the average time required to attain a Hb level of 10 g/dL or higher was found to be 9.69 ± 6.8 weeks for the DPO group, contrasting with 12.38 ± 7.33 weeks for the EPO group
Sinha et al. [49]	HD patients	Administering DPO at a lower dose frequency was equally effective and well tolerated as EPO in treating renal anemia in patients with ESRD undergoing dialysis.
Shoji et al. [50]	HD patients	DPO showed an advantage via higher potency in suppressing serum hepcidin-25 and promoting greater iron mobilization from body stores to the bone marrow compared to EPO.
Arrieta et al. [51]	HD patients	Switch from EPO to DPO resulted in a consistent and substantial 65% reduction in the required dose (p < 0.0001).
Hejaili et al. [52]		DPO demonstrated a favorable response in 78.6% of EPO-resistant patients, indicating its effectiveness in converting from high-dose EPO to DPO. Thus, DPO may offer cost savings for patients requiring high EPO doses, including those who do not respond well to EPO.

Based on the clinical trial data reflected in Table 3, DPO emerged as a superior and well-tolerated option for achieving and sustaining Hb levels, underscored by its reduced dosing frequency compared to EPO. DPO demonstrated advantageous outcomes over EPO in swiftly achieving target Hb levels while necessitating fewer dose adjustments. This advantage obviates the necessity for frequent monitoring, thereby positioning DPO as a favorable choice for anemia management. Other clinical studies conducted to evaluate the efficacy and safety of DPO in the management of anemia associated with CKD showed that DPO, administered at a reduced dose frequency of QW or biweekly demonstrated comparable efficacy and safety to r-HuEPO [45,46]. Table 4 describes other darbepoetin alfa clinical trial data.

**Table 4:** Summary of darbepoetin-alfa clinical trials rHuEPO = recombinant human erythropoietin; CAPD = continuous ambulatory peritoneal dialysis; HD = hemodialysis; PD = peritoneal dialysis; NA = not applicable; CKD = chronic kidney disease

Modality	No. of patients	Dosing schedule		Study conclusion
		Darbepoetin alfa	rHuEPO	
CAPD and HD [53]	69	Dose-escalation studies: 0.075–0.75 μg/kg/week	NA	DPO maintains Hb safely and effectively at 0.45 μg/kg once/week.
HD [54]	507	Once/week	3 times/week	DPO maintains Hb as effectively as rHuEPO.
HD and PD [55]	522	Once/week (n = 281), every other week (n = 66)	Once/week, twice/week, 3 times/week	DPO maintains Hb as effectively as rHuEPO.
HD and PD [56]	703	Once/week (n = 546), every other week (n = 157)	NA	DPO safely and effectively maintains Hb in patients switched from rHuEPO.
Dialysis [57]	122	Once/week (n = 90)	3 times/week (n = 31)	DPO is safe and effective when administered once/week.
HD and PD [58]	34	Every 3 weeks (n = 34)	NA	DPO maintains Hb safely and effectively when given every 3 weeks.
CKD, not yet requiring dialysis [59]	166	Once/week (n = 129)	Twice/week (n = 37)	DPO safely and effectively maintains Hb in patients with CKD who do not yet require dialysis.
CKD, not yet requiring dialysis [60]	23	Every other week (n = 23)	NA	DPO is effective when administered every 2 weeks for patients with CKD who do not yet require dialysis.

Safety profile comparison of darbepoetin alfa and erythropoietin alpha: insights from clinical trials in CKD-related anemia management

Several studies comparing the safety profiles of DPO and r-HuEPO in patients with CKD have shown similar incidences and compositions of adverse events. Studies conducted by Chen et al., Mehta et al., and Nissenson et al. have reported comparable safety profiles between DPO and EPO in terms of adverse events, changes in laboratory parameters and vital signs, and red blood cell transfusions [9,26,44]. These findings were consistent across various clinical trials conducted in both pre-dialysis and hemodialysis patients [9,44]. The common adverse events associated with DPO include infection (27%), hypertension (23%), hypotension (22%), myalgia (21%), headache (16%), and diarrhea (16%). Peripheral edema and fluid overload occurred at rates of 11% and 6%, respectively, while fatigue, fever, and influenza-like symptoms were reported at rates of 9%, 9%, and 6%, respectively. Adverse reactions observed in ≥5% of patients treated with EPO in clinical studies comprised hypertension (27.7%), arthralgia (16.2%), muscle spasm (7.4%), pyrexia (10.1%), dizziness (9.5%), vascular occlusion (8.1%), and upper respiratory tract infection (6.8%). The incidence rates highlight comparable safety profiles between DPO and EPO, with each demonstrating specific adverse effects within the observed percentages [9,26,43,44].

Patient-centric approach to anemia management: embracing darbepoetin alfa's reduced frequency of administration, clinical benefits, cost-effectiveness, and patient preference

Long-acting ESAs like DPO offer significant advantages over short-acting ESAs such as r-HuEPO in the management of anemia in CKD patients. While r-HuEPO requires frequent administration, often two to three times per week, due to its short half-life, long-acting DPO can be administered less frequently, typically once weekly or biweekly, improving patient adherence. This reduced dosing frequency is crucial for the long-term treatment required in CKD. For dialysis patients, switching to DPO significantly reduces the number of doses per year, enhancing compliance and reducing the risk of errors. Additionally, it lightens the workload of healthcare professionals and benefits pre-dialysis patients by offering greater convenience with less frequent hospital visits. This transition not only saves time and costs but also enhances overall patient satisfaction and quality of life.

Long-acting ESAs like DPO may initially have a higher cost compared to short-acting ESAs, but their potential for reduced dosing frequency and improved adherence can result in long-term cost savings by reducing healthcare resource utilization, hospital admissions, and transfusions. Cost modeling studies and investigations at HD centers have shown significantly lower annual costs for DPO, making it a cost-effective strategy. Considering factors like drug costs, administration frequency, and healthcare utilization, a comprehensive cost-effectiveness analysis supports DPO's clinical, social, and financial advantages over EPO in CKD anemia management.

Choosing darbepoetin alfa over erythropoietin alpha: considerations for optimal anemia management in clinical practice

**Figure 1:**
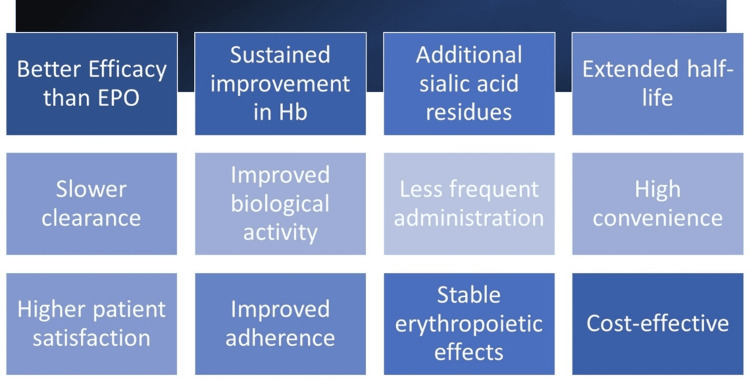
Features of darbepoetin alfa highlighting the superiority over erythropoietin alpha in anemia treatment for CKD patients Image credits: Abhishek T

Choosing between DPO and EPO for erythropoietic therapy in clinical practice involves considering factors like comparative efficacy, patient response, clinical objectives, dosing regimen, iron status, and patient preferences. Clinical guidelines, like those provided by KDIGO, offer valuable guidance for individualized ESA selection, dosing, and monitoring. A personalized approach, supported by clinical evidence, can optimize DPO utilization, given its pharmacokinetic advantages, reduced dosing frequency, and potential cost-effectiveness, improving treatment adherence and anemia management in CKD patients. Figure 1 depicts the unique advantages of DPO over EPO in the management of CKD anemia.

## Conclusions

In conclusion, DPO emerges as a promising solution for anemia management in CKD patients. Extensive clinical trials unequivocally demonstrate its superior efficacy, safety, and cost-effectiveness compared to EPO. DPO's favorable pharmacokinetics, reduced dosing frequency, and enhanced patient compliance make it a compelling choice, offering significant advancements in patient care and improving the quality of life for both predialysis and hemodialysis patients with CKD.
